# Acute Effect of L-Citrulline Supplementation on Resistance Exercise Performance and Muscle Oxygenation in Recreationally Resistance Trained Men and Women

**DOI:** 10.3390/jfmk8030088

**Published:** 2023-06-22

**Authors:** Adam M. Gonzalez, Yang Yang, Gerald T. Mangine, Anthony G. Pinzone, Jamie J. Ghigiarelli, Katie M. Sell

**Affiliations:** 1 Department of Allied Health and Kinesiology, Hofstra University, Hempstead, NY 11549, USA; yyang65@pride.hofstra.edu (Y.Y.); jamie.ghigiarelli@hofstra.edu (J.J.G.); katie.sell@hofstra.edu (K.M.S.); 2 Department of Exercise Science and Sport Management, Kennesaw State University, Kennesaw, GA 30144, USA; gmangine@kennesaw.edu; 3 Program in Exercise Science and Exercise Physiology, Kent State University, Kent, OH 44242, USA; apinzone@kent.edu

**Keywords:** nitric oxide, arginine, citrulline malate, ergogenic aid, resistance training

## Abstract

L-citrulline serves as a nitric oxide precursor with the potential to increase blood flow and improve resistance exercise performance, yet more research is needed to examine its ergogenic potential. To examine the effect of L-citrulline supplementation on resistance exercise performance, muscle oxygenation, and the subjective perception of effort, energy, focus, fatigue, and muscle pump, eighteen resistance-trained men (*n* = 11) and women (*n* = 7) (21.4 ± 1.8 years; 172.3 ± 7.5 cm; 76.9 ± 10.8 kg) were randomly assigned for supplementation with 8 g of L-citrulline (CIT) or a placebo (PL) in a cross-over fashion one hour prior to testing. Participants completed an isometric mid-thigh pull test (IMTP), a ballistic bench press protocol [two sets of two repetitions at 75% 1-repetition maximum (1 RM) with maximum ballistic intent], and a strength-endurance bench press protocol [five repetition-maximum sets at 75% 1RM]. Barbell velocity and power were measured via a linear position transducer during the ballistic protocol, while the repetitions completed, volume load and muscle oxygenation were quantified during the strength-endurance protocol. Subjective measures were assessed at the baseline and immediately pre- and post-exercise. Repeated measures of the analysis of variance and Bayesian equivalents revealed no significant interactions, providing evidence favoring the null hypothesis (BF10 < 1) for IMTP (PL 497.5 ± 133.6 vs. CIT 492.5 ± 129.4 N), barbell velocity, and power, and repetitions completed (PL 36.7 ± 7.2 vs. CIT 36.9 ± 8.1 repetitions). There were also no significant interactions for muscle oxygenation parameters or subjective measures except perceived fatigue. Women reported greater fatigue across all time points compared to men (~1.88 au, *p* = 0.045, BF10 = 0.2). The results indicate that a single 8 g dose of L-citrulline did not enhance isometric force production, muscle endurance, or muscle oxygenation parameters during the protocol implemented in this study.

## 1. Introduction

L-citrulline is a non-essential amino acid that is found primarily in watermelons, cucumbers, and other melons [1]. Due to its ability to be converted to L-arginine via the urea cycle, L-citrulline serves as a nitric oxide (NO) precursor with the potential to increase blood flow and augment resistance exercise performance [2]. When consumed, L-citrulline is converted to L-arginine for subsequent utilization by endothelial cells within the vessel walls to synthesize NO via the L-arginine-NO pathway [3]. L-citrulline has emerged as a better alternative to L-arginine supplementation because it can be well-absorbed and elevates L-arginine bioavailability and NO-dependent signaling to a greater extent compared to supplementing directly with L-arginine [4]. When L-arginine is supplemented directly, it is subjected to significant catabolism in the gut and through a first-pass metabolism which limits its systemic availability to promote NO synthesis. During resistance exercise, NO-mediated increases in vasodilation and blood flow could delay fatigue by allowing better oxygen and nutrient delivery to the working muscles to enhance strength and power expression and the replenishment of ATP between repeated resistance exercise sets [2]. For this reason, L-citrulline has garnered much attention as a potential performance-enhancing dietary supplement over the last decade [5], and it is currently among the most popular ingredients found in commercially available multi-ingredient pre-workout supplements, which are designed to improve exercise performance [6]. 

Several studies have investigated the effects of L-citrulline on strength performance, with most providing L-citrulline in the form of citrulline malate (a combination of L-citrulline and malate) [2]. Malate is an intermediate of the citric acid cycle that has been postulated to work synergistically with L-citrulline by upregulating oxidative ATP production during exercise, yet this lacks experimental evidence [7]. Citrulline malate is typically provided as an acute 8 g (g) dose in a 1:1 or 2:1 ratio which would yield approximately 4.0 to 5.3 g of L-citrulline and 3.7 to 4.0 g of malate. Some studies have shown that 8 g of citrulline malate could delay fatigue, improve power output, and increase the work performed during resistance exercise [8,9,10,11,12,13]. However, others have failed to demonstrate an ergogenic benefit following 8 g of citrulline malate [14,15,16,17,18,19]. Nevertheless, meta-analyses by Vårvik et al. [20] and Trexler et al. [21] observed improved repetitions to failure (6.4%; ~3 repetitions) and strength-power performance, respectively, in relation to high-intensity strength training and L-citrulline supplementation. Albeit both meta-analyses acknowledged that the effect size was small (Hedges’ G standardized mean difference = 0.196–0.20) and that more research was needed, particularly with higher doses. 

Research suggests that the effective dose of L-citrulline ranged from approximately 3 g up to possibly 10–15 g [1,5] since oral administration impacted L-arginine bioavailability in a dose-dependent manner (up to 15 g) without any adverse side effects [22]. Therefore, a higher L-citrulline dose may exert more robust enhancements in exercise performance. The purpose of this study was to examine the effect of supplementing with a higher dose of L-citrulline (8 g) on measures of strength, power, and strength-endurance. Additionally, muscle oxygenation parameters were measured to provide insight into the blood flow parameters during working sets and recovery. We hypothesized that L-citrulline supplementation could increase resistance exercise performance and augment muscle oxygenation compared to a placebo. 

## 2. Materials and Methods

### 2.1. Study Design

A randomized, crossover, placebo-controlled, double-blind study was employed to evaluate the effect of supplementation with 8 g L-citrulline on resistance exercise performance and muscle oxygenation in recreationally resistance trained men and women. Participants arrived at the Human Performance Laboratory for three sessions: one familiarization visit and two experimental trials. During the experimental trials, participants supplemented with either a placebo or 8 g L-citrulline one hour prior to completing assessments consisting of an isometric mid-thigh pull test (IMTP), a ballistic bench press protocol [2 sets of 2 repetitions at 75% 1-repetition maximum (1 RM) with maximum ballistic intent], and a strength-endurance bench press protocol [5 repetition-maximum (RM) sets at 75% 1RM]. Barbell velocity and power were measured via a linear position transducer during the ballistic protocol while repetitions completed, volume load, and muscle oxygenation were quantified during the strength-endurance protocol. Subjective measures were assessed at the baseline prior to supplementation (BL), pre-exercise (PRE), and immediately post-exercise (IP). 

Participants were instructed to continue their normal sleep, dietary, and exercise patterns throughout the data collection period but to avoid strenuous exercise and alcohol for 24 h prior to familiarization and the experimental trials. Experimental trials were scheduled for the same time of day between 7:00 and 9:00 a.m. and were separated by a 7-day washout period. For each experimental trial, participants were asked to arrive at the laboratory following an overnight fast (except for water). Participants were also asked to record their nutritional intake for the day prior to their first experimental trial and were then instructed to duplicate it as closely as possible for their subsequent experimental trial. In addition, participants were asked to refrain from consuming other dietary supplements throughout the duration of the study. 

### 2.2. Participants

Eighteen recreationally resistance-trained men (*n* = 11) and women (*n* = 7) volunteered to participate in this study. The descriptive characteristics of participants are presented in Table 1. A priori analysis (α = 0.05, β = 0.8), using a large effect size (Effect of *f =* 0.46) based upon changes in the total repetitions completed in previous work [23], indicated that at least 12 participants were required to detect significance. Participants were required to have at least one year of resistance-training experience, which included the bench press exercise. Exclusion criteria included any physical limitations, illnesses, or risk factors that could potentially limit exercise performance and/or the ingestion of medications or performance-enhancing drugs that could affect performance or exercise assessment. Following a thorough explanation of the study design and procedures, participants provided their written informed consent before participation in this study. The study was approved by the University Institutional Review Board prior to participant recruitment and data collection. 

### 2.3. One-Repetition Maximum Testing and Familiarization

During the familiarization session, participants’ height (±0.01 m), weight (±0.1 kg), and resting blood pressure were recorded. Blood pressure was recorded in duplicate and averaged using an automated blood pressure machine (OMRON 907XL Digital Blood Pressure Monitor, OMRON Healthcare, Inc., Lake Forrest, IL, USA). Next, participants were familiarized with an IMTP test (3 attempts) and assessed for a barbell bench press 1RM. The 1RM strength for the barbell bench press exercise was determined by applying a prediction formula based on the number of repetitions performed to fatigue using a fixed weight [24] and the methods previously described [25]. A certified strength and conditioning specialist monitored and enforced proper technique. After 1RM testing, participants were familiarized with the protocol of lifting with maximum ballistic intent in the bench press by pressing a standard unloaded barbell as rapidly as possible for a total of 2 repetitions, in accordance with previous work [23]. 

### 2.4. Supplementation

During each experimental trial, participants were provided with a 500 mL beverage one hour prior to completing a standardized dynamic full-body warm-up routine. The beverage contained either L-citrulline (CIT) or a placebo (PL). CIT contained 8 g of pure L-citrulline powder (Nutricost, Vineyard, UT, USA) mixed with non-caloric lemonade flavoring (Crystal Light^TM^, Kraft Foods, Inc. Chicago, IL, USA), while PL consisted of only non-caloric flavored water. The manufacturer provided a certificate of analysis verifying that the L-citrulline content of the dietary supplement was externally tested for accuracy. Both CIT and PL were served in disposable white plastic bottles and were indistinguishable in appearance and taste. The participants were blinded as to which supplement was provided during each trial, and supplements were mixed by a researcher not involved in data collection. Participants were randomized following block randomization. After consuming the supplement, participants underwent a one-hour seated rest period prior to initiating the warmup routine. 

The timing of supplement ingestion was consistent with most of the existing literature examining the ergogenic effect of L-citrulline on resistance exercise. L-citrulline has been shown to reach peak plasma concentrations ~42 min following oral administration [4,22]. The dosage, however, is greater than previous investigations examining the effect on resistance exercise performance, which have provided L-citrulline in the form of 8 g citrulline malate, which yields 4–5.3 g L-citrulline [20]. 

### 2.5. Isometric Mid-Thigh Pull 

The IMTP was included to assess total body muscular strength using methods previously described [25]. During each test, participants stood on force plates and were instructed to assume an athletic stance with the barbell at the height of and in contact with the mid-thigh. The participant’s barbell setting was recorded at the familiarization visit and used for both experimental trials. On a “go” command, participants were instructed to pull upwards on the barbell as hard and fast as possible and to maintain the maximal effort for four seconds. The force output was recorded using a force plate (Vernier Software and Technology, Beaverton, OR USA) sampling at 1000 Hz and a data collection computer using Logger Pro 3 software (Vernier Software and Technology). The force plates were positioned in a standardized location and calibrated prior to each pull. During each experimental trial, participants completed three pulls, which were interspersed with a rest period of three minutes. The highest peak force (N) value among the three sets was used for analysis. The IMTP protocol was in accordance with previous work demonstrating high reliability [26].

### 2.6. Ballistic Bench Press Protocol

Prior to completing the bench press protocols, participants completed a bench press-specific warm-up consisting of 8 repetitions at 40% 1RM and 4 repetitions at 60% 1RM, which were separated by a two-minute rest period. The ballistic bench press protocol consisted of 2 sets of 2 repetitions at 75% 1RM with maximum ballistic intent separated by a 3 min rest period [23,25]. Concentric barbell velocity (m·s^−1^) and power (W) were measured via a linear position transducer (Tendo Power Output Unit, Trencin, Slovak Republic). This device was placed in a standardized location adjacent to the rack and with the cord attached to the lateral portion of the barbell collar. The peak power, mean power, and mean velocity were recorded for each repetition and then averaged for each set. The highest average value across both sets was identified for statistical analysis. Our group demonstrated an excellent test–retest reliability with the device (ICC_3,1_ = 0.91). 

### 2.7. Strength-Endurance Bench Press Protocol

Following a 5 min rest period, participants completed 5 RM sets using 75% 1RM with 2 min of rest between each set. The sets were concluded when the participant could not complete another repetition without assistance or with proper technique. The total repetitions completed and volume load (kg·repetitions) were recorded for each set and used for statistical analysis. All experimental trials were overseen by the same certified strength and conditioning specialist. 

### 2.8. Muscle Oxygenation 

Muscle oxygenation parameters were assessed using a near-infrared spectroscopy (NIRS) muscle oxygen sensor (MOXY Muscle Oxygen Monitor, Hutchinson, MO, USA) in accordance with procedures and analysis from previous work [25,27]. During the 5 min rest period prior to completing the strength-endurance bench press protocol, a MOXY device was attached to the participant’s dominant arm on the muscle belly of the anterior deltoid at the midpoint between the clavicle and the insertion of the deltoid on the humerus using a MOXY adhesive bandage to hold the device in place and shield from external light. The device monitored tissue oxygenation parameters by emitting infrared light into the blood vessels of the region over which it was attached using a modified Beer–Lambert Law to analyze the amount of light absorbed at wavelengths pertaining to oxygenated and deoxygenated hemoglobin. This device provided the concentration of oxygenated hemoglobin (HbO_2_) relative to the total hemoglobin (tHb), output for the muscle oxygen saturation [SmO_2_ (%) = HbO_2_/tHb]. The MOXY monitor has shown excellent validity and reliability for assessing oxygen saturation measurements compared to mixed venous oxygen saturation monitoring [28,29,30]. 

Three variables were utilized to examine muscle oxygenation dynamics during the strength-endurance bench press protocol. The first was the percent change in SmO_2_ (∆%SmO_2_) from the start (SmO_2_start) to the end (SmO_2_stop) of each set. SmO_2_start was recorded as the SmO_2_ value one second before each bench press set began, while SmO_2_stop was recorded as the SmO_2_ value at the end of the concentric phase of the final repetition of each set. Equation (1) was used to calculate Δ%SmO_2_ [31]:

(1) $$\Delta \%SmO_{2}=\left(\left(\frac{SmO_{2}stop\times 100}{SmO_{2}start}\right)-100\right)\times -1$$

The second value was the muscle oxygen resaturation rate (SmO_2_RecSlope). This variable was measured as the slope of SmO_2_ values for 30 s immediately following the final repetition of each bench press set. The third variable was SmO_2_Peak. This variable was recorded as the highest measured SmO_2_ value achieved during each recovery period between sets. The starting and ending SmO_2_ values, ∆%SmO_2_, SmO_2_RecSlope, and SmO_2_Peak were maintained for statistical analysis. A graphical depiction of these variables can be found in Gonzalez et al. [25].

### 2.9. Subjective Feelings and Ratings of Perceived Exertion

Questionnaires were provided at BL, PRE, and IP to assess the subjective feelings of focus, energy, and fatigue using a 15 cm visual analog scale. Questions were structured as “My level of focus is:”, and a 15 cm line was anchored by the words “low” and “high” to represent extreme ratings. Participants were asked to rate their feelings at each time point by marking on the corresponding line. The validity and reliability of the visual analog scale were previously established [32]. RPE for the bench press protocol was assessed via the OMNI weightlifting scale [33] after the completion of the strength-endurance bench press protocol to evaluate the participant’s level of effort. A modified session RPE score where the RPE value was multiplied by the total repetitions completed was also calculated to quantify perceived effort relative to the work completed.

### 2.10. Statistical Analysis 

Data were modeled using both a frequentist and Bayesian approach. The frequentist approach involved two-tailed, two- (sex × condition), and three-way (sex × condition × time [Sets 1–5; or BL, PRE, and IP]) analyses of variance (ANOVA) with repeated measures for dependent variables measured at each time point. The Greenhouse-Geisser adjustment was applied to degrees of freedom when sphericity was violated. Significant interactions and their main effects were further examined using Bonferroni’s post hoc analysis. The assumption of normality was verified by a Shapiro–Wilk test. The criterion alpha was set at *p* ≤ 0.05. To further assess the likelihood (or the effect of condition and/or time) of the data under an alternative hypothesis compared to the null hypothesis, the Bayesian equivalent of each of these tests was performed with default prior scales [34]. The likelihood was represented in the form of Bayes factors (i.e., BF_10_) and was interpreted according to the recommendations of Wagenmakers et al. [35]. That is, data were interpreted as evidence in favor of the null hypothesis when BF_10_ < 1. Otherwise, they were interpreted as “*anecdotally*” (1 < BF_10_ < 3), “*moderately*” (3 < BF_10_ < 10), “*strongly*” (10 < BF_10_ < 30), “*very strongly*” (30 < BF_10_ < 100), or “*extremely*” (BF_10_ > 100) in favor of the alternative hypothesis. All statistical analyses were performed using JASP 0.14.1 (Amsterdam, The Netherlands). All data were reported as the mean ± SD. 

## 3. Results

### 3.1. Dietary Compliance and Adverse Effects

All participants verified their duplicated nutritional intake for the day prior to each experimental trial. No adverse effects were reported. 

### 3.2. Muscular Performance

**Figure 1:**
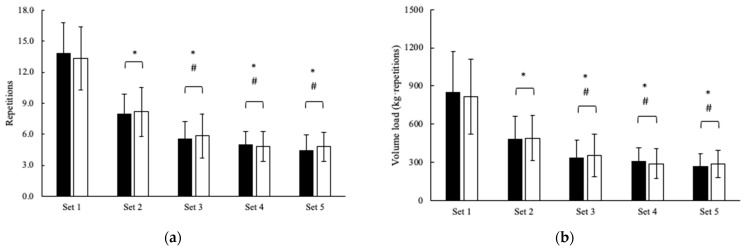
Effect of L-citrulline on five sets of the bench press for (**a**) Repetitions and (**b**) Volume load completed (mean ± SD). Note: PL = black bars; CIT = open bars; * = Significantly (*p* < 0.001) different from set 1; # = Significantly (*p* < 0.001) different from set 2.

The performance differences during the IMTP and bench press protocols between conditions are presented in Figure 1 and Table 2. No differences were seen between the conditions for performance during the IMTP and ballistic bench press protocol (see Table 2). Evidence favored the null hypothesis that no differences existed between PL (36.7 ± 7.2 repetitions) and CIT (36.9 ± 8.1 repetitions) in the total repetitions completed (*p* = 0.746; BF_10_ = 0.3) across five sets of the bench press. Likewise, no differences between PL (2239 ± 736 kg·repetitions) and CIT (2234 ± 739 kg·repetitions) were seen with a total volume load (*p* = 0.914; BF_10_ = 0.2). Only anecdotal evidence was found for the main effects of time (*p* < 0.001, BF_10_ = 1.0), where all subsequent sets were significantly lower than sets 1 and 2 (see Figure 1). The IMTP, peak and mean power and total volume load were significantly (*p* < 0.05) and expectedly higher in men, though evidence still favored the null hypothesis (BF_10_ ≤ 1.0).

### 3.3. Muscle Oxygenation

**Figure 2:**
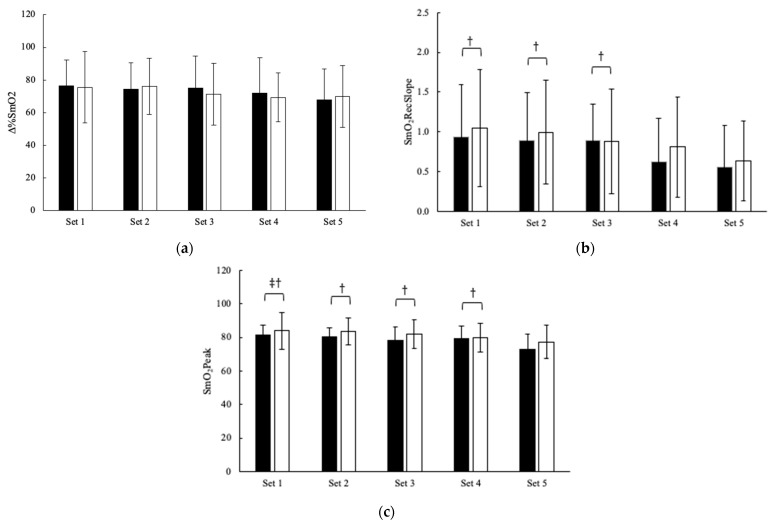
Effect of citrulline on five sets of bench press for (**a**) ∆%SmO2, (**b**) SmO2RecSlope, and (**c**) SmO2Peak (mean ± SD). Note: PL = black bars; CIT = open bars; ‡ = Significantly (*p* < 0.05) different from set 4. † = Significantly (*p* < 0.05) different from set 5.

Changes in muscle oxygenation during the bench press protocol are illustrated in Figure 2. Aside from observing the significant (*p* < 0.001) main effects for time, evidence favored the null hypothesis (BF_10_ = 0.1) for SmO_2_RecSlope and SmO_2_Peak. Compared to the first three sets, SmO_2_RecSlope was lowest in set 5 (*p* < 0.05), whereas the SmO_2_Peak significantly (*p* < 0.05) declined in set 5 compared to sets 1 through 4. Set 4 was also significantly reduced from set 1 (*p* = 0.044) for the SmO_2_Peak. No other differences were seen between the conditions or sex in measures of muscle oxygenation.

### 3.4. Subjective Assessments

Subjective assessments of perceived effort, energy, focus, fatigue, and muscle pump are presented in Table 3. Although evidence favored the null hypothesis for all comparisons using each subjective perception variable (BF_10_ ≤ 1.0), the significant main effects for sex (*p* < 0.05) were noted for fatigue and muscle pump, with women reporting higher values in each. Additionally, fatigue was specifically higher in women throughout PL but not CIT. Otherwise, the main effects for time (*p* < 0.001) were noted for focus, fatigue, and muscle pump. A greater focus was observed at PRE compared to BL, as well as at IP compared to BL and PRE, while greater fatigue was seen at IP compared to BL and PRE. For muscle pump, a sex × time interaction (*p* = 0.003) was seen where women reported a greater pump at BL and PRE (*p* < 0.05) but not at IP. Meanwhile, for both men and women, during each condition, muscle pump was the same at BL and PRE before increasing at IP.

## 4. Discussion

This study examined the effect of acute L-citrulline supplementation on resistance exercise performance, muscle oxygenation, and the subjective measures of perceived effort, energy, focus, fatigue, and muscle pump. No differences were observed between the conditions for peak force during the IMTP, ballistic bench press power and velocity, or the total repetitions and volume completed during the strength-endurance bench press protocol. Additionally, no differences were seen between conditions for measures of muscle oxygenation and perception. The results of this study showed that a single 8 g dose of L-citrulline did not enhance isometric force production, bench press performance, or muscle oxygenation parameters in recreationally resistance trained men and women. 

Despite administering a higher dose in comparison to the current body of literature (8 g L-citrulline vs. 8 g citrulline malate containing ~4–5.3 g L-citrulline), our findings agree with several studies that have failed to demonstrate an acute ergogenic benefit following L-citrulline supplementation on muscle endurance during high-intensity strength training. Gonzalez et al. [18] reported no improvement in the number of repetitions performed during a protocol consisting of five sets of bench press at 75% 1RM with 2 min rest intervals following 8 g of citrulline malate. Similarly, studies from Chappell and colleagues showed that 8 g of citrulline malate did not increase the repetitions completed during 10 sets of isokinetic knee extensions at 70% of peak concentric force output [14] or 10 sets of barbell curls at 80% 1RM [15]. Farney et al. [16] also showed that 8 g of citrulline malate did not improve the performance or rate of fatigue during leg extensions following the completion of a high-intensity exercise circuit consisting of squats, lunge jumps, squat jumps, and lateral jumps. Lastly, Trexler et al. [19] failed to show a benefit from 8 g of citrulline malate on performance during an isokinetic dynamometer protocol consisting of five sets of 30 maximal effort single-leg extensions. 

Conversely, several studies have shown that acute L-citrulline supplementation could improve resistance exercise performance by improving the number of repetitions performed before reaching voluntary muscular failure over several sets of multi-joint exercises. Studies from Wax and colleagues have shown that 8 g of citrulline malate could improve the repetitions performed during a protocol consisting of five sets of leg presses, hack squats, and leg extensions at 60% 1RM [13] and during a protocol consisting of three sets of bodyweight chin-ups, reverse chin-ups, and push-ups [12]. An acute 8 g dose of citrulline malate was also reported to improve repetitions of failure during eight sets of bench press at 80% 1RM [11] and during six sets of bench press and leg press exercises at 80% 1RM [10]. Therefore, the current state of evidence is mixed, yet a recent meta-analysis [20] showed how citrulline malate could significantly increase repetitions to failure during high-intensity strength training. Although speculative, one potential reason why L-citrulline did not exert an ergogenic effect in the current study could be due to the limited activation of musculature in the upper body. Studies involving multi-joint lower body exercises have shown to result in a high tendency for a performance effect [20].

L-citrulline’s role in promoting the relaxation of the vascular smooth muscle may positively impact blood flow to the working muscle during exercise. However, in the current study, no differences were observed between CIT and PL trials for measures of muscle oxygenation. While evidence has supported that L-citrulline supports NO-dependent signaling [4] and endothelial-dependent vasodilation 60 min following ingestion at rest [36], only a few studies have examined markers of blood flow and muscular perfusion surrounding resistance exercise. In agreement with our findings, these studies failed to report outcomes indicative of an improved blood flow following a bout of resistance exercise [18,19,25,37]. Cutrufello et al. [37] showed no effect of 8 g of citrulline malate on flow-mediated dilation of the brachial artery following five sets of machine chest presses at 80% 1RM. After the acute administration of 8 g of citrulline malate, Trexler et al. [19] also showed no enhancement of the femoral artery diameter and blood flow or vastus lateralis cross-sectional area following an isokinetic dynamometer knee extension protocol. Similarly, Gonzalez et al. [18] reported no enhancement in the triceps brachii muscle thickness following five sets of bench press at 75% 1RM. Lastly, following a similar protocol to the current study, 7 days of supplementation with watermelon juice containing 2.2 g L-citrulline also failed to augment brachial artery diameter or muscle oxygenation parameters during the bench press protocol [25]. 

This study was not without its limitations. First, the small sample size, the inclusion of fewer women, and the use of healthy, recreationally resistance-trained individuals limit our ability to generalize to the whole population. We also did not assess plasma citrulline, arginine, or NO bioavailability to validate the pharmacokinetic effects of the higher dose of L-citrulline. Next, this study administered a single acute dose of L-citrulline. Based upon some of the existing evidence on endurance performance, it has been postulated that chronic dosing (e.g., >7 days) may be more effective for enhancing exercise performance [5]. However, the intent of this study was to compare against previous interventions, which administered a single acute dose of 6–8 g citrulline malate. Additionally, given the potential for L-citrulline to induce NO-mediated increases in vasodilation, allowing for better oxygen and nutrient delivery to the working muscles, it was also possible that the protocol implemented in the current study did not induce enough fatigue to observe the benefit of L-citrulline. 

## 5. Conclusions

Many studies have examined the effect of L-citrulline, typically in the form of 8 g of citrulline malate, on weight training repetition performance along with strength and power outcomes. While these findings have been mixed, L-citrulline supplementation has been associated with an increase in strength-endurance, and high-intensity strength and power performance [20,21]. Our data suggest that a single 8 g dose of L-citrulline did not enhance the isometric force production, muscle endurance, or muscle oxygenation parameters during the protocol implemented in this study. Future research should continue to examine the efficacy of different acute and chronic doses of L-citrulline on strength and power performance and other forms of high-intensity training.

## Figures and Tables

**Table 1:** Descriptive characteristics of participants.

Characteristic	Men	Women
Age (y)	21.4 ± 2.0	21.4 ± 1.9
Height (cm)	176.4 ± 6.3	165.9 ± 3.7
Body mass (kg)	83.0 ± 8.4	67.3 ± 6.1
Resting systolic blood pressure (mmHg)	128.2 ± 6.6	114.6 ± 10.7
Resting diastolic blood pressure (mmHg)	70.3 ± 7.0	72.9 ± 6.6
Resistance training experience (y)	5.1 ± 2.5	4.6 ± 2.1
Bench press 1RM (kg)	98.7 ± 16.9	58.6 ± 14.3
Relative strength (1RM/Body mass)	1.2 ± 0.2	0.9 ± 0.2

**Table 2:** Strength and power differences between sexes and conditions (mean ± SD).

				Sex		Condition		Sex × Condition	
		PL	CIT	*p*	BF_10_	*p*	BF_10_	*p*	BF_10_
Isometric Mid-Thigh Pull									
Peak force (N)	Women	366 ± 44	362 ± 54	<0.001	1.0	0.523	0.0	0.894	0.2
	Men	581 ± 97	576 ± 85						
Ballistic Bench Press									
Peak Power (W)	Women	359 ± 84	359 ± 75	<0.001	1.0	0.151	0.0	0.161	0.8
	Men	625 ± 168	689 ± 204						
Mean Power (W)	Women	241 ± 39	234 ± 36	<0.001	1.0	0.382	0.0	0.144	0.5
	Men	413 ± 100	438 ± 106						
Mean Velocity (m·sec^−1^)	Women	0.58 ± 0.11	0.56 ± 0.08	0.602	0.6	0.455	0.5	0.085	0.4
	Men	0.58 ± 0.11	0.62 ± 0.14						

**Table 3:** Perceptional differences between conditions (mean ± SD).

		BL		PRE		IP	
RPE (au)							
PL	Women	N/A		N/A		8.0 ± 0.6	
	Men					8.6 ± 0.8	
CIT	Women					8.1 ± 0.7	
	Men					8.4 ± 1.0	
Session RPE (au)							
PL	Women	N/A		N/A		310 ± 61	
	Men					307 ± 77	
CIT	Women					320 ± 64	
	Men					295 ± 76	
Focus (au)							
PL	Women	8.2 ± 2.0		9.9 ± 1.4	*	10.9 ± 1.4	*
	Men	9.9 ± 3.5		10.9 ± 3.4		11.4 ± 3.1	
CIT	Women	8.3 ± 2.0		9.7 ± 1.5		10.2 ± 1.5	
	Men	9.9 ± 3.3		11.1 ± 2.5		11.9 ± 2.4	
Energy (au)							
PL	Women	8.7 ± 1.6		9 ± 1.1		9.3 ± 2.4	
	Men	9.1 ± 3.5		10.4 ± 3.3		6.9 ± 4.2	
CIT	Women	8.0 ± 2.0		9.5 ± 1.3		8.8 ± 2.8	
	Men	9.2 ± 3.7		9.5 ± 4.4		8.8 ± 3.8	
Fatigue (au)							
PL ^$^	Women	6.2 ± 1.3		6.2 ± 1.8		8.3 ± 2.8	*,#
	Men	3.5 ± 2.4		2.4 ± 2.5		6.2 ± 4.5	
CIT	Women	5.8 ± 2.8		4.8 ± 2.3		6.9 ± 2.7	
	Men	3.5 ± 2.7		2.5 ± 2.6		8.9 ± 4.8	
Muscle pump (au)							
PL	Women	5.3 ± 2.8	$	5.6 ± 3	$	10 ± 1.9	#
	Men	1.5 ± 1.5		1.5 ± 1.7		10.7 ± 2.9	#
CIT	Women	5.4 ± 3.3		5.1 ± 3		11 ± 2	#
	Men	1.3 ± 1.5		1.5 ± 1.5		11.2 ± 2.9	#

BL = baseline prior to supplementation; PRE = pre-exercise; IP = immediately post exercise; * = Significantly (*p* < 0.001) different from BL; # = Significantly (*p* < 0.001) different from PRE; $ = Significantly (*p* < 0.05) different between men and women; au = arbitrary units.

## Data Availability

The data presented in this study are available upon request from the corresponding author.
